# Genetic Basis Analysis for Candidate QTLs and Functional Genes Controlling Four-Seeded Pods at Lower-Node in Soybean (*Glycine max*) Plant

**DOI:** 10.3390/plants15060966

**Published:** 2026-03-20

**Authors:** Ramiz Raja, Yihan Huang, Shicheng Ning, Bo Hu, Mahfishan Siyal, Wen-Xia Li, Hailong Ning

**Affiliations:** Key Laboratory of Soybean Biology, Ministry of Education, Northeast Agricultural University, Harbin 150030, China; rajahussain497@gmail.com (R.R.); huangyihan2027@163.com (Y.H.); mehfishan131@gmail.com (M.S.)

**Keywords:** soybean, four seedpod numbers in lower part, QTL, candidate genes, haplotype analysis

## Abstract

Soybean (*Glycine max* L. Merr.) is a globally significant oilseed crop. The number of four-seeded pods in the lower part (FSPL) serves as a critical yield component under high-density planting. To date, numerous crop-specific traits have been investigated in multiple breeding studies of soybean; however, little attention has been paid to studies on FSPL. Hence, in this study, we investigated the genetic basis of FSPL using a recombinant inbred line population (RIL3613) across four environments. The segregated genetic mapping population was cultivated during the field experiments, and the collected phenotypic dataset of FSPL exhibited quantitative genetics and high broad-sense heritability (0.724), indicating stable genetic control. Further, we performed quantitative trait locus (QTL) mapping using raw means in each environment and identified 10 QTL, explaining phenotypic variations (PVE) ranging from 0.10% to 2.94%. Among the identified environmentally stable QTL, *qFSPL-15-1* was consistently detected across all environments. Two candidate genes [*Glyma.15G034100* (encoding lysophosphatidic acid acyltransferase 2) and *Glyma.15G034200* (encoding an RNA-binding protein)] were predicted within the flanking genomic interval. The allele frequencies of haplotype combinations of Hap1: Pro2 + CDS1 for *Glyma.15G034100* and Hap3: Pro3 + CDS1 for *Glyma.15G034200* in wild soybeans (26.6–30.0%) were larger than improved cultivars (52.6–53.4%). We believe that our current findings elucidate the molecular mechanisms regulating lower-pod formation and provide precise genetic targets for marker-assisted selection in high-yield soybean breeding.

## 1. Introduction

Soybean (*Glycine max*) is an important source of edible protein (40%) and oil (20%) for use in food, feed, and industry. With the growing global demand driven by advancements in food processing technologies, enhancing soybean seed yield has become a primary breeding goal [1,2]. Generally, soybean seed yield is improved through contributing traits such as seed weight, pod number per plant, seed number per plant, and seed number per pod [3].

Among the qualitative and quantitative traits of soybean, the FSPN is of particular importance as it significantly affects total seed production when total pod number per plant exhibits limited variation, and increasing the number of FSPN becomes a vital strategy to enhance yield [4]. When grown under higher-density condition, the number of pods at the lower portion is prone to be affected and vary greatly, thereby impacting the overall yield [5,6]. Lower-node pods often initiate and begin filling earlier in the reproductive period than higher-node pods, which can result in a longer seed-filling duration and greater assimilate accumulation per pod, contributing more consistently to final seed yield. This suggests that retention of lower-node four-seeded pods could have a unique role in yield stability, particularly under variable environmental conditions. However, further targeted studies are needed to quantify this effect explicitly [7]. Therefore, the genes that control the formation of the lower four pods are of great significance for improving the stability of yield.

With the objective of molecular marker-assisted breeding, quantitative trait locus (QTL) mapping has been conducted on pod number related traits. To date, over 400 QTLs related to pod number have been identified using various populations, such as the recombinant inbred lines (RILs) and F_2_ generation [8,9,10]. Among these, 86 QTLs associated with FSPN were mapped using RIL and F_2_ populations [5,11]. The genetic mechanism of pod distribution in soybean is uneven across the plant and its vertical structure. Previous studies have focused mostly on genetic mechanisms that promote FSPN growth in the whole plant, while little attention was paid to the four-seed pod number in lower part [12,13]. Although the four-seeded pod number (NFSP) is recognized as an important yield component in soybean, relatively few studies have focused specifically on this trait compared to broader yield component traits [14]. For example, an FSPN QTL had been fine-mapped and some candidate genes had been identified. A genome-wide association study of seed number per pod acknowledged the seed number trait, but noted limited reports targeting NFSP exclusively [14,15].

In order to explain the formation of FSPN under varied conditions, genetic pathways have been investigated [16], for example, calcium signal pathways, and calcium and ABA signaling have been shown to interact at multiple levels in plant stress responses, with calcium acting as a second messenger and ABA modulating transcriptional and stress response pathways [17]. In legumes such as soybean, regulatory networks involving ABA and calcium signal pathways have been identified as influencing reproductive development, including pod formation and expansion [18]. However, the specific connection between these signaling systems and four-seed pod number (FSPL) remains largely underexplored in the context of yield component genetics and requires deeper investigation. Compared with the number of mapped QTL, studies on genes controlling the formation of four pods per plant are relatively limited. The *Ln* gene controls leaf shape and thereby affects the ratio of the four pods [19]. The gene *GmCYP78A10* could regulate the proportion of four-seeded pods, which is a member of the P450/CYP78A family [20].

In the recent era, multi-omics approaches have made the feasibility for rapid genotyping and identification of candidate genetic loci as well as genes controlling qualitative and quantitative traits in agricultural crops. To dissect the complex genetic architecture of FSPL trait, high-resolution genotyping is a critical prerequisite. Compared to lower-density arrays such as SoySNP50K or traditional PCR-based markers, the SoySNP660K array offers significantly higher marker density, which includes more than 660,000 SNPs across the soybean genome, enabling the precise delineation of recombination breakpoints and the detection of rare allelic variations. This high-density genotyping facilitates the construction of a fine-scale genetic map and allows for the accurate identification of QTLs associated with traits like FSPL. By averaging this platform, large genomic gaps are minimized and statistical power is enhanced across multiple environments.

To achieve a higher seed yield, it is essential to improve seed yield in soybean. It is crucial to explore the genetic variations in seed pod numbers FSPL associated with different positions on the plant. This study hypothesizes that identifying stable quantitative trait loci +QTLs for FSPL across multiple environments will provide insights into the genetic factors controlling pod set and seed filling. The objectives of this study are to identify stable QTLs for FSPL in soybean across four different environments, nine candidate genes regulating FSPL, and examine haplotypic differences in FSPL-related genes among wild soybeans, landraces, and improved cultivars. These results are expected to enhance our understanding of the genetic basis of FSPL and contribute to molecular breeding strategies for improving soybean yield.

## 2. Results

### 2.1. Phenotypic Variation Analysis on Four-Seed Pod Numbers in the Lower Part of the Soybean Plant

The significant genotype variance and range (from minimum to maximum) showed extensive genetic variation in FSPL among all individuals of RIL3613. This indicates the population is favorable for mapping QTL for FSPL. The larger values of kurtosis and skewness showed that multiple genes might control FSPL. The significant genotype-by-environment (G × E) interaction observed, along with the differences in average, standard deviation, kurtosis, and skewness across environments, indicates that the expression of genes controlling FSPN was influenced by environmental factors. QTL analyses were conducted separately for each environment, and environment-specific QTLs were identified, highlighting the role of (G × E) interactions in the regulation of FSPL. The higher broad-sense heritability observed over multiple environments suggests the stable effects of common genes (Table 1 and Table 2). Multi-environment QTL analysis confirmed that these genes contribute consistently to the trait, although environmental variation influenced the expression of some QTLs.

### 2.2. QTL Controlling Four-Seed Pod Numbers in the Lower Part of the Soybean Plant

A total of 10 QTLs controlling FSPL were detected on seven chromosomes, with each individual QTL explaining between 0.10% and 2.94% of the phenotypic variation (Table 3). In 2019, 2020, 2021 and 2022, 5, 3, 2 and 5 additive QTLs were detected, respectively, with PVE of 10.92%, 2.38%, 0.83% and 2.32%, respectively. Through the analysis of epistatic effects, a total of 312 QTL pairs were detected, with the variation range of PVE being 0.20% to 2.05% (Table S1). In 2019, 2020, 2021, and 2022, 166, 41, 27, and 88 pairs of interacting QTLs were detected, respectively, with PVE of 71.44%, 33.26%, 37.71%, and 59.93%, respectively. These small PVE values suggest a polygenic architecture for FSPL, where multiple small-effect QTLs contribute to the trait’s overall phenotypic variation. This further indicates the complex genetic control of FSPL, with numerous loci exerting diffident individual effects.

The additive effects of these QTL were all in a negative direction, which showed that the allelic variant of these QTL from Dongnong L13 (female parent) could reduce FSPL and those from Heihe 36 (male parent) could enhance FSPL. Among these, three QTLs were consistently identified across multiple environments, indicating their stability. Stable *qFSPL-15-1* identified in four environments, showed the largest PVE of 2.94% in 2019.

### 2.3. Prediction of Candidate Genes Associated with FSPL

A total of 43 genes were identified within the *qFSPL-15-1* interval (Table S1). Among these 43 genes, seven genes showed nonsynonymous single-nucleotide variations, and five genes, *Glyma.15G031700*, *Glyma.15G033800*, *Glyma.15G033900*, *Glyma.15G034100*, and *Glyma.15G034200* exhibited different variation patterns between the two parents (Table S2). Further screening was carried out by comparing gene annotations, *Glyma.15G033800 and Glyma.15G033900* were excluded due to the lack of functional annotation (Table S1).

**Figure 1:**
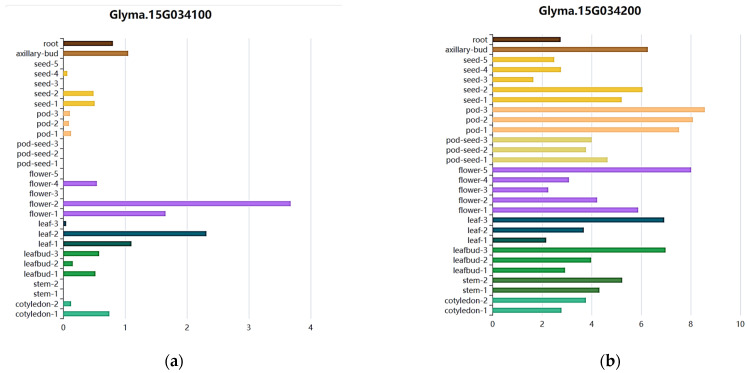
Expression level of *Glyma. 15G034100* (**a**) and *Glyma 15G034200* (**b**) in various tissues.

Based on gene expression levels in different tissues, *Glyma.15G031700* was also excluded because it had extremely low expression. *Glyma.15G034100* is annotated as lysophosphatidic acid acyltransferase 2 (LPAAT2). The association between LPAAT2 and four-seeded pod number (FSPL) is likely to be indirectly mediated via lipid metabolism: phosphatidic acid (PA) synthesized by LPAAT2 is both a key intermediate in lipid biosynthesis and a signaling molecule regulating phytohormone pathways related to ovule and pod development. Expression data retrieved from the SoyOmics soybean multi-omics database https://ngdc.cncb.ac.cn/soyomics/index (accessed on 15 September 2025) revealed that LPAAT2 is expressed during the early stages of seed and pod development (Figure 1a), supporting its important role in seed development and seed germination. *Glyma.15G034200* gene is annotated as a member of the RNA-binding (RRM/RBD/RNP motif) family of proteins, which play important roles in regulating gene expression, RNA processing, and responding to environmental signals. Expression data revealed that *Glyma.15G034200* is highly expressed in both seeds and pods (Figure 1b).

Conserved domain prediction was performed for the proteins encoded by the two candidate genes *Glyma.15G034100* and *Glyma.15G034200* using the NCBI CD-Search tool https://www.ncbi.nlm.nih.gov/Structure/cdd/wrpsb.cgi (accessed on 2 March 2026) against the Conserved Domain Database (CDD). A highly conserved PLN02380 domain was identified in the protein encoded by *Glyma.15G034100*. Additionally, two tandem RNA Recognition Motif superfamily (RRM_SF) domains were detected in the protein encoded by *Glyma.15G034200*. The expression patterns suggested that during the early stages of seed development, *Glyma.15G034100* and *Glyma.15G034200* may play an important role in pod number. Therefore, these two genes are hypothesized to be closely related to the pod-number regulatory pathway, thereby promoting FSPL.

### 2.4. Haplotype Analysis of Candidate Genes

We did not identify any SNPs mutation in promoter regions, while a nonsynonymous mutation was detected in the CDS region. Based on the sequencing data and the phenotypic data of FSPL of 95 individuals in the RIL3613 population, haplotype analysis was conducted for the candidate genes.

**Figure 2:**
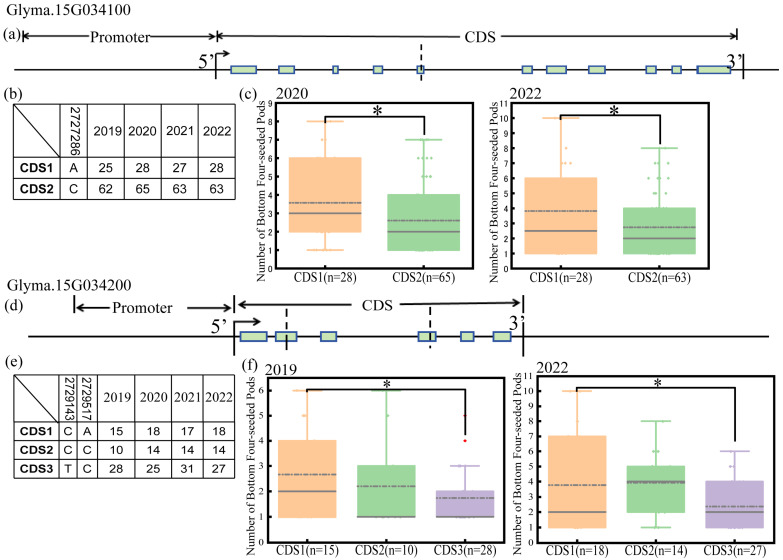
Haplotype analysis of the coding regions of the *Glyma.15G034100* and *Glyma.15G034200* genes in the RIL3613 population across four environments (2019–2022). (**a**) Mutation information of the *Glyma.15G034100* gene. (**b**) The number of the two haplotypes of the *Glyma.15G034100* gene in four environments as determined by sequencing. (**c**) Haplotype analysis of the *Glyma.15G034100* gene in the CDS region. The height of the bars represents FSPL. (**d**) Mutation information of the *Glyma.15G034200* gene. (**e**) The number of the three haplotypes of the *Glyma.15G034200* gene in four environments as determined by sequencing. (**f**) Haplotype analysis of the *Glyma.15G034200* gene in the coding region. * represent significant difference at *p* < 0.05 using *t-*test.

However, a SNP mutation at position of 2,802,998 bp in the exon region of the *Glyma.15G034100* gene was found, which causes the base to change from A to C and the encoded amino acid to change from histidine to proline. By this SNP mutation, the gene is divided into two haplotypes, namely CDS1 and CDS2 (Figure 2a,b). The phenotype of FSPL was compared between different haplotypes of *Glyma.15G034100*. The results showed that there were significant differences (*p* < 0.05) between the two haplotypes in two different environments (2020 and 2022). The average FSPN of CDS2-type individuals was significantly lower than that of CDS1, indicating that the specific variation in the CDS region of this gene may be related to the change in FPSL (Figure 2c).

There were two SNP mutations in the exon region of *Glyma.15G034200* gene, causing the bases to change from C to T and from A to C at the positions of 2,809,462 and 2,809,835 bp, respectively, which caused the encoded amino acid to change from proline to leucine, and from serine to proline. Based on these two SNP mutations, this gene can be divided into three haplotypes, namely CDS1, CDS2, and CDS3 (Figure 2d,e).

By comparing the FSPN phenotype of different haplotypes of *Glyma.15G034200*, the averages of the two haplotypes showed significant differences in two distinct environments (2019 and 2022) at *p* < 0.05. Among them, the average of FSPL of the CDS3-type individuals is significantly lower than that of the CDS1 type, indicating that the specific variation in the CDS region of this gene may be related to FSPL change (Figure 2f). As shown in the figure, CDS1, CDS2 and CDS3 represent different haplotypes in the coding region of this gene; The *t*-test was used to evaluate the differences between groups, where * indicates *p* < 0.05.

### 2.5. Variation in the Cis-Regulatory Elements of Promoter Among Candidate Genes Haplotypes

Further, we found a sequence variation in the promoter region of the *Glyma.15G034100* gene among the 238 soybean germplasm resources in Northeast China. To investigate whether these mutations cause changes in the cis-regulatory elements and thereby affect the expression level of the gene, we used the Plant CARE website https://bioinformatics.psb.ugent.be/webtools/plantcare/html/ (accessed on 18 December 2025) to predict the cis-regulatory elements of the promoter of the three haplotypes of the *Glyma.15G034100* gene, and used the TBtools 1.0 software for visual analysis.

**Figure 3:**
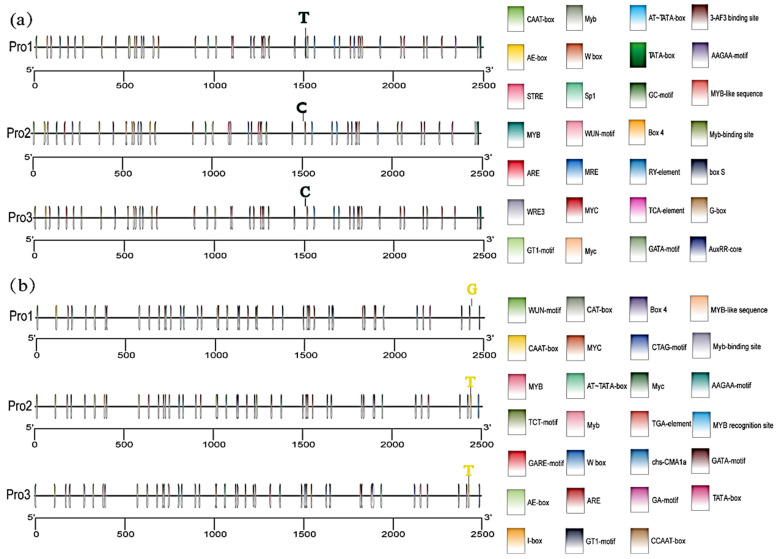
Analysis of cis-acting elements in the promoters of *Glyma.15G034100* and *Glyma.15G034200* genes. (**a**) Cis-acting element map of the *Glyma.15G034100* gene promoter. (**b**) Cis-acting element map of the *Glyma.15G034200* gene promoter.

The results showed that the C base at position 986 bp upstream of the gene was mutated to a T base, resulting in an additional TATA-box cis-regulatory element in the promoter of the *Glyma.15G034100* gene (Figure 3a). The TATA-box, a core cis-regulatory element, exhibited an increased copy number in the promoter region. This may enhance the efficiency of transcription initiation for the corresponding gene, which could potentially influence agronomic traits such as pod number by modulating the transcription of pod-related genes and resource allocation within the plant.

A sequence variation in the promoter region of the *Glyma.15G034200* gene was also found. Using the same method for analysis as above, the T base at position 68 bp upstream of this gene was mutated to a G base, resulting in the loss of a CAAT-box in the promoter of the *Glyma.15G034200* gene (Figure 3b). In plants, the polymorphism of the CAAT-box may indirectly influence agronomic traits such as pod number and plant height, possibly by modulating the expression of genes associated with plant growth, development, and reproduction.

### 2.6. Variation in Physicochemical Properties and Tertiary Structure in Protein Among Candidate Gene Haplotypes

A nonsynonymous A-to-C substitution at position 2,802,998 bp in the exon of *Glyma.15G034100* resulted in an amino acid change from histidine to proline (H377P). To explore the potential functional impacts of this substitution, we first mapped the H377P site to its conserved domain. The results showed that H377P is localized within the PLN02380 conserved domain (spanning amino acids 96–447), which serves as the core catalytic region of 1-acyl-sn-glycerol-3-phosphate acyltransferase (LPAAT)—a key enzyme in lipid biosynthesis.

The wild-type amino acid sequence was retrieved from the Phytozome database https://phytozome-next.jgi.doe.gov/ (accessed on 14 December 2025), and the mutated sequence was generated using the NovoPro translation tool V2.5. https://www.novopro.cn/tools/translate.html (accessed on 14 December 2025). Physicochemical property prediction via Expasy ProtParam 2020 https://web.expasy.org/protparam/ (accessed on 15 December 2025) revealed differences in isoelectric point, extinction coefficient, instability index, and grand average of hydropathicity (GRAVY) between the two haplotypes (Table 4).

**Figure 4:**
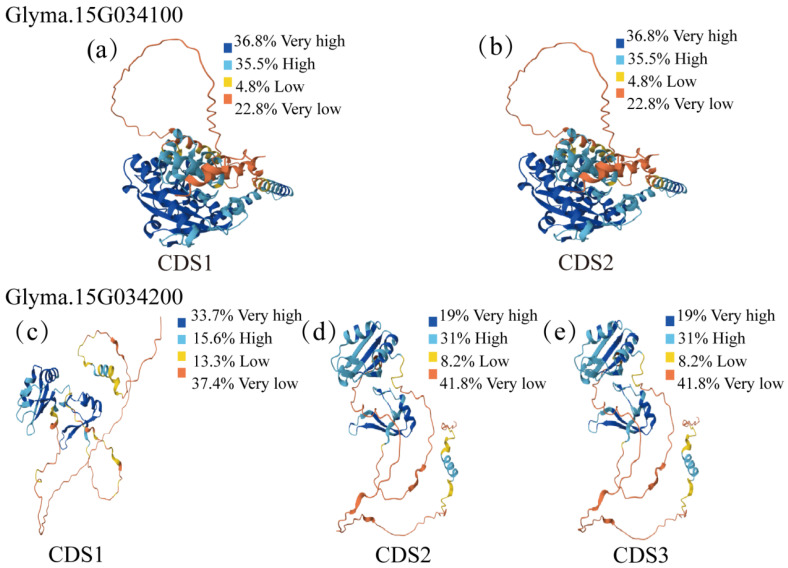
Tertiary structure diagrams of proteins encoded by different genotypes of *Glyma.15G034100* and *Glyma.15G034200* genes. Note: (**a**,**b**) are the tertiary structure diagrams of proteins encoded by CDS1 and CDS2 genotypes of *Glyma.15G034100* gene; (**c**–**e**) are the tertiary structure diagrams of proteins encoded by CDS1, CDS2 and CDS3 genotypes of *Glyma.15G034200* gene, respectively.

Tertiary structures of the wild-type and mutated proteins were predicted using AlphaFold3 https://alphafoldserver.com/ (accessed on 16 December 2025), and the models exhibited high overall consistency. Notably, high-confidence regions (pLDDT > 70) spanned amino acids 92–426, indicating that the functional domain of the protein is likely enriched in this segment (Figure 4). To quantify structural divergence, we calculated the Root Mean Square Deviation (RMSD) between the aligned structures of the two haplotypes, which was only 0.005 Å—far below the widely accepted 0.5 Å threshold for significant conformational changes. This finding implies that the H377P substitution did not induce substantial alterations in the core protein architecture. Collectively, these results suggest that although H377P is localized within the PLN02380 core catalytic domain of LPAAT and does not cause marked tertiary structure changes RMSD=0.005 Å, the observed differences in physicochemical properties (e.g., isoelectric point, instability index) may modulate the enzyme’s catalytic activity or substrate binding capacity. In turn, this could regulate lipid metabolism and downstream reproductive development pathways, ultimately contributing to variations in four-seeded pod number (FSPL) in soybean.

Two nonsynonymous mutations were identified in the exon of *Glyma.15G034200*: An A C-to-T substitution at 2,809,462 bp (proline-to-leucine, P290L) and an A-to-C substitution at 2,809,835 bp (threonine-to-proline, S328P). We first mapped the mutation sites to the protein’s conserved domains. Results showed that the S328P, P290L, and their combined substitutions were all localized to the flanking regions outside the two RNA Recognition Motif (RRM) domains (2–60 and 101–172 aa).

Physicochemical property prediction of proteins encoded by the three haplotypes (CDS1, CDS2, CDS3) revealed differences in instability index and grand average of hydropathicity (GRAVY) among the three haplotypes (Table 4).

Tertiary structures of the three haplotype-encoded proteins were predicted using AlphaFold3 https://alphafoldserver.com/ (accessed on 16 December 2025). Notable structural differences were observed among CDS1-, CDS2-, and CDS3- encoded proteins, with minimal divergence between CDS2 and CDS3. High-confidence regions (pLDDT > 70) of the CDS1-encoded protein were concentrated at 1–61 and 101–172 aa, while those of CDS2 and CDS3 were located at 1–61 and 98–172 aa (Figure 4).

To quantify structural divergence, pairwise structural alignment and Root Mean Square Deviation (RMSD) calculation was performed:

Hap1 vs. Hap2 (S328P substitution): RMSD = 0.009 Å

Hap2 vs. Hap3 (P290L substitution): RMSD = 0.005 Å

Hap1 vs. Hap3 (combined P290L/S328P substitutions): RMSD = 0.012 Å

All RMSD values were far below the widely accepted 0.5 Å threshold for significant conformational changes, indicating that the substitutions did not induce substantial alterations in the core protein architecture.

Collectively, these results suggest that although the P290L and S328P substitutions of *Glyma.15G034200* are localized in the RRM domain flanking regions and do not cause marked tertiary structure changes (all RMSD < 0.012 Å), the observed differences in physicochemical properties (e.g., instability index, hydropathicity) may modulate protein folding, stability, or protein–protein interactions. These changes could further affect the protein’s post-transcriptional regulatory function, ultimately contributing to soybean four-seeded pod number (FSPL) variation.

#### Superior Allele Genotype in the Germplasm Resources

**Figure 5:**
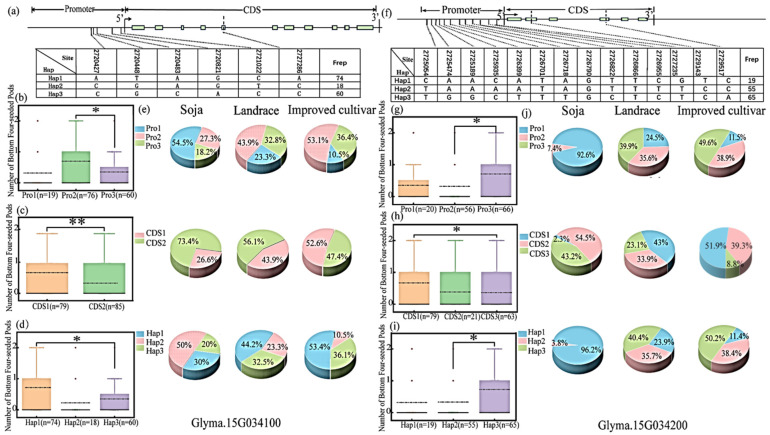
Haplotype analysis of *Glyma.15G034100* and *Glyma.15G034200* genes in the germplasm population and their distribution among varieties of different evolutionary types. (**a**) Mutation information of *Glyma.15G034100* gene. (**b**) Haplotype analysis on promoters of *Glyma.15G034100* gene. (**c**) Haplotype analysis on CDS of *Glyma.15G034100* gene. (**d**) Haplotype analysis on Haps of *Glyma.15G034100* gene. (**e**) Proportions of different Pro, CDS and Hap haplotypes of *Glyma.15G034100* gene in wild soybean, landraces and improved cultivars. (**f**) Mutation information of *Glyma.15G034200* gene. (**g**) Haplotype analysis on promoters of *Glyma.15G034200* gene. (**h**) Haplotype analysis on CDS of *Glyma.15G034200* gene. (**i**) Haplotype analysis on Haps of *Glyma.15G034200* gene. (**j**) Proportions of different Pro, CDS and Hap haplotypes of *Glyma.15G034200* gene in wild soybean, landraces and improved cultivars. Note: The height of the column indicates the number of 4-seeded pods at the basal node of the plant; different colors represent different sample groups. Pro1/Pro2/Pro3 refer to three haplotypes of the gene promoter, CDS1/CDS2 refer to two haplotypes of the coding region, and Hap1/Hap2/Hap3 refer to three combined haplotypes derived from the joint analysis of the promoter and coding region (Hap1: CDS1 + Pro2, Hap2: CDS2 + Pro1, Hap3: CDS2 + Pro3). The differences between groups were evaluated by the t-test, where * indicates *p* < 0.05 and ** indicates *p* < 0.01.

Among the 238 soybean germplasm resources in Northeast China, the *Glyma.15G034100* gene has a mutation of five bases in the promoter region, which can be classified into three haplotypes, namely Pro1, Pro2 and Pro3. There is also an SNP mutation in the exon region, causing the histidine at position 377 to be changed to proline. This gene can be divided into two haplotypes, namely CDS1 and CDS2. Based on the variations in *Glyma.15G034100* gene in the promoter and exon regions, there are a total of three combinations (Hap1: CDS1 + Pro2, Hap2: CDS2 + Pro1, Hap3: CDS2 + Pro3), named Hap1, Hap2 and Hap3 (Figure 5a).

Among them, the average of FSPL of the Pro-2-type individuals was significantly greater than that of the Pro3 type; the average of FSPL of the CDS1-type individuals was significantly greater than that of the CDS2 type; and the average of FSPL of the Hap1 type was significantly greater than that of the Hap3-type individuals. Therefore, the Pro2, CDS1 and Hap1 types are excellent haplotypes for FSPL in soybeans (Figure 5b–d).

Using the re-sequencing data of 2883 varieties in the GPP from the National Biomedical Information Center https://ngdc.cncb.ac.cn/soyomics/index (accessed on 17 December 2025), the proportions of different haplotypes in wild soybeans, local varieties, and improved varieties were analyzed. The frequencies of the superior haplotypes Pro2, CDS1 and Hap1 of *Glyma.15G034100* were 0.0%, 27.5% and 0.0% in wild soybeans, 41.6%, 43.2% and 47.9% in landraces, and further increased to 50.0%, 52.6% and 51.1% in improved cultivars, respectively (Figure 5e). A chi-square test of independence $\chi^{2}-test$ indicated that the frequencies of the three superior haplotypes showed a significant upward trend from wild soybeans to improved cultivars, with significant or extremely significant differences in distribution among populations $Pro2:\chi^{2}=10.8762,p<0.01;CDS1:\chi^{2}=16.8923,p<0.001;Hap1:\chi^{2}=14.9524,p<0.001$. This result suggests that the proportions of the elite haplotypes Pro2, CDS1 and Hap1 increased in a statistically significant manner during domestication and breeding, implying that breeders may have directionally retained these haplotypes in the breeding process due to their potential association with the four-seeded pod numbers in the lower section of the soybean plant.

The *Glyma.15G034200* gene has 12 base mutations in the promoter region, which can be classified into three haplotypes: Pro1, Pro2, and Pro3. There are two SNP mutations in the exon region, causing the proline to change to leucine at position 230 and the Threonine to Proline at position 289. Depending on the two SNP mutations in the exon region, this gene can be divided into three haplotypes: CDS1, CDS2, and CDS3. Based on the variations in the *Glyma.15G034200* gene in the promoter and exon regions, there are a total of three combinations (Hap1: Pro1 + CDS3, Hap2:Pro2 + CDS2, Hap3:Pro3 + CDS1), and they are named Hap1, Hap2, and Hap3 (Figure 5f).

Among them, the average of FSPL of the Pro3-type individuals was significantly greater than that of the Pro2 type; the average of FSPL of the CDS1-type individuals was significantly greater than that of the CDS3 type; and the average of FSPL of the Hap3 type was significantly greater than that of the Hap2 type. Therefore, the Pro3, CDS1 and Hap3 types are excellent haplotypes for FSPL in soybeans (Figure 5g–i).

In the GPP, the proportions of different haplotypes in wild soybeans, local varieties, and improved varieties were analyzed. The superior haplotypes Pro3, CDS1 and Hap3 of *Glyma.15G034200* were present at extremely low frequencies in wild soybeans (0.0%, 2.3% and 0.0%, respectively). Their proportions increased significantly to 39.9%, 43.0% and 40.4% in landraces, and further rose to 49.6%, 51.9% and 50.2% in improved cultivars (Figure 5j). The chi-square test of independence revealed extremely significant differences in the distribution of the three superior haplotypes among different soybean populations $Pro3:\chi^{2}=40.4341,p<0.001;CDS1:\chi^{2}=89.7141,p<0.001;Hap3:\chi^{2}=39.7202,p<0.001$. The above data demonstrate that the proportions of these elite haplotypes increased statistically significantly during the evolution of soybean from wild to cultivated and then to improved cultivars, with the highest proportions observed in improved cultivars. This phenomenon is likely related to the selection and retention of excellent agronomic traits associated with FSPL during the breeding process.

## 3. Discussion

### 3.1. The Polygenic Architecture of FSPL

FSPN is a key indicator of seed quality and yield, and an increased FSPN could significantly enhance soybean seed yield [22,23]. Previously, various genes and QTLs related to FSPN in the whole plant have been identified [8,9,24,25]. Considering the difference in influence of environment on the FSPN, the QTL mapping had been conducted on FSPN in two bi-parent RILs [13] and a four-way RIL [12], respectively.

Summarizing the above studies and the present one, three genetic characters indicated that FSPL was a typical quantitative trait. The first is the continuous phenotypic distribution. The second is the moderate heritability. The heritability was 0.4461–0.5296 in single-plant conditions [12] and 0.724 across multiple environment (Table 2). The third is that fewer QTLs could be detected which can explain less phenotypic variations. A total of 4 and 13 QTLs with PVE of 0.03–0.64 [13], 9 QTLs with PVE of 3.63–10.81 [12], and 10 QTLs PVE of 0.10–2.94 (Table 3) were detected. The quantitative nature of FSPL is clearly established, with multiple QTLs contributing to its variation. However, the small PVE values observed (0.10–2.94%) suggest a polygenic architecture for FSPL, where numerous small-effect loci collectively regulate the trait. These findings indicate that FSPL is a complex trait, and while a significant number of QTLs have been identified, their individual effects are modest. The small PVE values further reinforce the idea that the genetic control of FSPL involves many genes with subtle contributions, highlighting the challenge of identifying large-effect loci in polygenic traits.

Summarizing previous studies and our current work, three empirically confirmed genetic characteristics demonstrate that FSPL is a typical quantitative trait: (1) a continuous phenotypic distribution in segregating populations; (2) moderate to high heritability, with values of 0.4461–0.5296 under single-plant conditions [12] and 0.724 across multiple environments in our study (Table 2); (3) phenotypic variation is controlled by multiple small-effect loci, as supported by 4 and 13 QTLs (PVE 0.03–0.64%) [13], 9 QTLs (PVE 3.63–10.81%) [12] in previous reports, and 10 QTLs (PVE 0.10–2.94%) identified in our study (Table 3).

These confirmed results clearly establish the quantitative nature of FSPL. Based on these findings, we infer that FSPL has a polygenic genetic architecture, where numerous small-effect loci collectively regulate the trait. The consistently low PVE of individual QTLs further supports that the genetic control of FSPL relies on many genes with subtle effects, highlighting the challenge of identifying large-effect loci for this polygenic trait.

### 3.2. The Co-Location of QTL Underlying FSPL

Among the 10 additive QTLs identified, five have been located in previous studies. *qFSPL-2-1, qFSPL-2-2, qFSPL-9-1* and *qFSPL-16-2* were located within the intervals of seed set 5-3 (Gm02: 97983-47042650) [13], qPod number1-1 (Gm02: 4961320-9327815) [21], qPod number-K-4 (Gm09: 9265135-20552886) [12] and qPN-J-6 (Gm16: 10498970-26989225) [12], respectively. The interval of qFSPL-17-1 partially overlapped with those of qPN-D2-2 (Gm17:9974328-24327803), qPN-D2-4 (Gm17: 9949907-13150237) and qPN-D2-6 (Gm17: 9088800-9949918) [12], and the gene *Glyma.17G128900* (Gm17:10330413-10331258) was located in this interval, which regulates the number of four-seeded pods [26]. The other five QTLs were discovered in this research and need to be verified in subsequent studies.

### 3.3. The Basis of Candidate Genes Selection

In this research, a stable QTL *qFSPL-15-1* was mapped in four environments, while this QTL showed modest explanatory power. This may come from the polygenic architecture of FSPL with numerous small-effect loci, limited population size and recombination events, unmodeled epistatic interactions, or the influence of unlinked regulatory networks not captured by additive QTL models.

Based on the nonsynonymous single-nucleotide variations, functional annotation, and gene expression analysis, *Glyma.15G034100*, annotated as lysophosphatidic acid acyltransferase 2 (LPAAT2) isozyme in *Arabidopsis* (*Arabidopsis thaliana*), ensures that developing seeds and pods receive the necessary nutrients and energy for fertilization and pod formation. This is achieved through its crucial role in triacylglycerol (TAG) biosynthesis, which provides energy reserves during seed and pod development [27]. Moreover, by regulating hormonal signaling, such as auxin and gibberellin pathways, carbon allocation, and stress tolerance, LPAAT2 directly influences pod distribution and seed filling, which are crucial for optimal soybean yield [28]. The enhanced lipid accumulation through LPAAT2 also supports seed viability and pod set under environmental stress conditions [28]. Therefore, LPAAT2 has a main role in improving pod fertility and early pod set in soybean, providing valuable insights for breeding programs aimed at increasing soybean productivity and resilience to environmental stress [29,30,31,32]. And *Glyma.15G034200*, a member of the RNA-binding protein family, was found to be highly expressed in both seeds and pods, suggesting its potential involvement in regulating FSPL. Several RNA-binding proteins, play major roles in *Arabidopsis* such as GLUTAMINE-RICH PROTEIN23 (GRP23) and various embryo-defective (EMB) proteins, as well as maize EMP4, EMP5, PPR8522, and rice OGR1, which are known to play key roles in regulating the translation of key genes involved in embryo and kernel development [33,34,35]. These proteins may function by binding to specific mRNA sequences, controlling their stability and translation, and thus modulating main processes like hormone signaling, stress responses, and developmental control in a tissue or node manner [36,37,38].

Haplotype analysis was used to determine the relative expression levels of candidate genes in the two parents, Heihe-36 and Dongnong-L13 across RIL3613 population. Haplotype analysis revealed an SNP at position 2,722,679 and 2,729,144 bp on chromosome 15, where an A-to-C substitution defined two haplotypes: *Glyma.15G034100*-Hp1 (A allele), *Glyma.15G034100*-Hp2 (C allele). And *Glyma.15G034200*-Hap1 (CT), *Glyma.15G034200*-Hap2 (CC), and *Glyma.15G034200*-Hap3 (AC). Across four environments, lines carrying Glyma.15G034100-Hp2 consistently exhibited reduced FSPL compared with *Glyma.15G034100*-Hp1, suggesting that *Glyma.15G034100*-Hp1 represents a favorable haplotype for increasing basal four-seed pod number. These findings provide essential molecular evidence for marker-assisted selection in soybean, identifying *Glyma.15G034100* and *Glyma.15G034200* as candidate genes for functional validation and breeding applications. In this study, the nonsynonymous mutations among haplotypes (H377P in *Glyma.15G034100*, S328P and P290L in *Glyma.15G034200*) did not significantly alter protein 3D conformation, as indicated by very low RMSD values. However, we cannot rule out linked genetic variations in the chromosomal regions of the target haplotypes, including regulatory elements, intronic polymorphisms, or other functional genes within the same LD block. Given the minimal structural changes, these mutations are unlikely to affect protein function. Thus, the phenotypic differences among haplotypes are more likely caused by linkage drag from adjacent regions rather than the missense mutations themselves. In future breeding, high-density markers, fine mapping, or gene editing will be used to reduce linkage drag and introgress only the favorable haplotypes. Moreover, fine genetic mapping, gene functional validation and expression analyses and CRISPR-Cas9 are required to verify the role of the predicted genes in this study. The differential expression patterns of these genes during key developmental stages underscore their potential role in pod number regulation, offering a promising avenue for enhancing soybean yield and production through targeted genetic manipulation.

## 4. Materials and Methods

### 4.1. Research Materials

The material from the soybean plant was included in a linkage panel designated as RIL3613. The linkage panel included 120 RILs derived from a cross of two soybean cultivars, Dongnong L13 (with FSPL of 1) and Heihe 36 (with FSPL of 7). The crossing occurred in Harbin, and the F_1_ offspring seeds were harvested the following year (2008). Seeds were sown in Harbin from 2010 to 2014 (located at (126.63° E). And (45.75° N) in the summer and winter in Yacheng (109.00° E and 17.50° N) (Table 1). The offspring of each generation were selected using the single-seed selection method. Then, an RIL population consisting of 120 lines was formed and was given the name RIL3613, with the complete procedure described in [13]. The population used for haplotype analysis included 164 improved varieties which are all spring sowing type soybean.

The soybean germplasm population (GPP) was strictly divided into three subpopulations according to germplasm origin and evolutionary history: wild soybean (Glycine soja), landraces, and improved cultivars. Wild soybeans are undomesticated germplasms from natural habitats, landraces are traditional cultivated soybeans without modern breeding improvement, and improved cultivars are developed via modern breeding techniques to enhance key agronomic traits.

### 4.2. Field Experiments and Phenotypic Data Collection

The whole lines in population RIL3613 were planted in Acheng Experimental and Practical Training Base of Northeast Agricultural University (127.63° E) and (45.82° N) in 2019, 2020, 2021 and 2022. All the varieties for haplotype analysis were planted in 2022. The experiment employed a randomized complete block design with three replications. All the lines are sowed in density of 22 × 10^4^ plants/hm^2^, and the dose of N, P_2_O_5_, K_2_O fertilizer are 18, 46, 30 kg/hm^2^, respectively. Seeds were planted in ridges of 3 m in length and 0.67 m in width.

The field experiments were managed according to the frequently applied agronomic methods for growing soybean crop. A random selection of 10 plants from each ridge determined the number of pods on each plant. On the plant, the number of pods was determined in the upper, middle, and lower parts [39]. The plot’s observed value was calculated using the mean of 10 plants, while the observed values of the three replications were averaged upper (U), middle (M), and lower based on the total number of nodes. If the total number of nodes was exactly divisible by three (3X), each part (U, M, L) limited X nodes. If the number of nodes was 3X + 1, the extra pods were allocated to the middle portion, and the distribution of X:X + 1:X. If the number of pods was 3X + 2, one extra node was added to the upper and one to the lower part. Total pod numbers per plant were also calculated across all node positions. The mean value of 10 plants represented the plot data, and the averages across three replications were used as the phenotypic value for QTL mapping. The vertical distribution of pod number traits, including nodes bearing one to four-seed pods, was analyzed separately in the upper, middle, and lower parts of the plant.

### 4.3. Phenotypic Variation Analysis

Based on the average of three replications, the descriptive statistical parameters, including means, coefficients of variation, kurtosis, skewness, and normality assessments were calculated. The analysis of variance (ANOVA) for multiple environments was conducted using SAS software (Version 9.2; The SAS Institute, Cary, NC, USA). Broad-sense heritability was evaluated using the following model.

$$h^{2}=\frac{\sigma_{G}^{2}}{\sigma_{G}^{2}+\frac{\sigma_{GE}^{2}}{e}+\frac{\sigma_{\epsilon }^{2}}{re}}$$

where $h^{2}$ represents the broad-sense heritability of phenotypic trait, $\sigma_{G}^{2}$ denotes the genotypic variance, $\sigma_{GE}^{2}$ represents the genotype-by-environment interaction variance, and $\sigma_{\epsilon }^{2}$ represents the error variance. The parameter *e* indicates the number of environments, while *r* refers to the number of replications.

### 4.4. Bin Marker Map and QTL Analysis

We employed the SoySNP660K BeadChip array (Beijing Boao Biotechnology, Beijing, China) [31], a high-density genotyping platform that provides comprehensive genome-wide coverage of single nucleotide polymorphism (SNP) markers using the RIL3613 population. After quality screening of large sequencing data with the criteria of a maximum allele frequency > 0.05 sites per SNP and maximum missing sites < 10%, a total of 108,342 SNPs was discovered in 20 linkage groups. To determine the potential cross points for the RIL3613 SNPs, The SNP binner software for Python 2.7 https://github.com/solgenomics/SNPbinner (accessed on 25 January 2026) was used. The minimum distance between cross points in base pairs was 15 SNPs. After that, the aggregate breakpoints that were derived from the cross points for the entire population were employed to produce the representative bins (the minimum size of each bin is 100 kb), which resulted in 2177 bins. These markers were anchored on 20 linkage groups covering 3539.66 cM in a linkage map using QTL ICI Mapping version 4.1 (https://www.isbreeding.net/ (accessed on 25 January 2026)) [40].

The means of three replications of FSPL phenotype in each environment were used to QTL mapping. The detection of additive and epistatic QTLs was accomplished using inclusive competitive interval mapping via BIP function in QTL ICI Mapping software (version 4.10). For additive and epistatic QTL mapping, the genome-wide scan step was set to 1.00 cm, the logarithm of odds (LOD) threshold was 2.5 and 5.0, respectively, and the *p*-value for entering variables (PIN value) was 0.001.

The QTLs were named using the earlier reported method of McCouch et al. [41].

### 4.5. Identification of Potential Candidate Genes

Potential candidate genes were identified using the Phytozome website (https://phytozome.jgi.doe.gov/ (accessed on 25 January 2026)) in the QTL repeatedly detected in multiple environments. Then the genes with the nonsynonymous single-nucleotide variations between two parents were screened. The third selection was carried out by comparing gene annotations information and protein function in the NCBI database (https://www.ncbi.nlm.nih.gov/ (accessed on 25 January 2026)) and pathway analysis in the Kyoto Encyclopedia of Genes and Genomes (KEGG) (https://www.kegg.jp/ (accessed on 25 January 2026)). The fourth step was to screen the genes expressed in the stem tissues. After the above screen, the rest of the genes may play an important role in regulating pod number in the lower part of the soybean plant.

### 4.6. Haplotype Analysis

A verification population (VP) consisting of 164 varieties was re-sequenced and the haplotypes of each objective gene were determined by comparing single-nucleotide variations. Primers were designed to target the coding sequence (CDS) regions containing missense mutation sites of candidate genes. Based on exon variations in the coding sequence (CDS) regions observed in the sequencing data, two haplotypes (Hap1 and Hap2), were defined.

Haplotype analysis of the candidate genes was then conducted by associating the haplotype variations with the phenotypic data for FSPL across the tested lines in every environment solely. Then the differences in FSPL between haplotypes were tested by Haploview 4.2. (https://haploview.software.informer.com/4.2/ (accessed on 25 January 2026)). The haplotype association analysis was conducted as the following statistical model was:

*p_ij_* = *u* + *hap_i_* + *e_ij_*

where is the phenotype of FSPL of *i*th allelic genotype in the *j*th line, and is the genetic effect expect *i*th allelic gene and error effect for *j*th line. *p* = phenotypic value, u = over all population mean of the trait, hap = haplotype, e = Random residual error associated with the j-th individual carrying haplotype i.

### 4.7. Analysis of the Structure of Candidate Gene Promoter Elements

A re-sequencing of 238 soybean germplasm resources from Northeast China was conducted to analyze sequence variations in the promoter regions of candidate genes. Promoter sequence alignment was performed using DNAMAN software (Version 10.0). To investigate whether these mutations led to changes in cis-regulatory elements and thereby affected the expression levels of the genes, the cis-regulatory elements of the promoters of different haplotypes of the candidate genes were predicted using the PlantCARE website https://bioinformatics.psb.ugent.be/webtools/plantcare/html/ (accessed on 18 December 2025), and visual analysis was performed using the TBtools 1.0 software.

### 4.8. Analysis of Physicochemical Properties and Tertiary Structure Predictions of Protein of Candidate Gene Haplotypes

In order to investigate whether the exon mutations of candidate genes lead to changes in the physicochemical properties and tertiary structure of the proteins, thereby affecting the regulatory role of the genes on the FSPL. The amino acid sequences of the candidate genes were downloaded from the phytozome website https://phytozome-next.jgi.doe.gov/ (accessed on 14 December 2025).

The mutated amino acid sequences were calculated using the website https://www.novopro.cn/tools/translate.html (accessed on 14 December 2025) The physicochemical properties of the protein sequences of different haplotypes were analyzed using the Expasy Protparam website https://web.expasy.org/protparam/ (accessed on 15 December 2025), and the differences in isoelectric point, extinction coefficient, instability coefficient, and average hydrophobicity of different haplotypes were predicted. The tertiary structure of the protein sequences of different haplotypes was predicted using the alphafold3 website https://alphafoldserver.com/ (accessed on 16 December 2025). To quantify structural divergence, pairwise structural alignment was performed, and the Root Mean Square Deviation (RMSD) was calculated accordingly. The three-dimensional structures of the proteins were modeled using the Swiss-Model server https://swissmodel.expasy.org/ (accessed on 2 March 2026), and subsequent structural alignment and RMSD calculation were conducted using PyMOL software (Version 3.1.6.1).

## 5. Conclusions

In this research, ten QTLs associated with FSPL of soybean were detected, and two genes, *Glyma. 15G034100 and Glyma.15G034200*, were mined in an environmentally stable QTL *qFSPL-15-1*. The superior haplotypes of the mutation of the promoter and exon regions Hap1: Pro2 + CDS1 for *Glyma.15G034100* and Hap3: Pro3 + CDS1 for *Glyma.15G034200* showed variation among the evolutionary type of the wild, landraces and improved cultivars of soybean. This study reveals the genetic basis of FSPL and provides theoretical and technical support for its molecular breeding on the high-yield soybean cultivars.

## Figures and Tables

**Table 1:** A descriptive analysis of four-seed pod numbers in the lower part of the soybean plant.

Environment	Parents		RIL3613									
	Dongnong L13	Heihe 36	Average	Standard Deviation	Minimum	Maximum	Kurtosis	Skewness	Correlation			
									2019	2020	2021	2022
2019	1	7	1.89	1.61	1	10	7.07	2.48	-	0.54	0.17	−0.03
2020	1	8	2.73	2.04	1	10	0.76	1.24	0.54	-	0.19	0.06
2021	1	7	2.84	2.14	1	11	1.78	1.37	0.17	0.19	-	0.19
2022	1	6	2.86	2.31	1	10	0.99	1.35	−0.03	0.06	0.19	-

“-“ represent no correlation.

**Table 2:** Analysis of variance (ANOVA) and estimation of heritability on four-seed pod numbers in the lower part of the soybean plant.

Source	DF	SS	MS	F	Pr > F	*σ* ^2^
Environment (Block)	8	1.014	0.127	1.62	0.1143	
Environments (E)	3	176.601	58.867	753.54	<0.0001	
Genotypes (G)	119	3218.541	27.047	346.22	<0.0001	1.668
G × E interaction	347	2644.903	7.622	97.57	<0.0001	2.520
Error	932	72.808	0.078			0.078
Total variation	1409	6155.966				
Heritability						0.724

DF, degrees of freedom; SS, sum of squares; MS, mean square; F, F-statistic; Pr > F, probability of obtaining a larger F value by chance; σ^2^, variance component estimates.

**Table 3:** Summary of detected additive QTLs controlling four-seed pod number in the lower part of RIL3613 mapping population of soybean.

QTL	Chromosome	Left Marker	Right Marker	Physical Region		Environment	LOD	PVE (%)	Additive Effect	LeftCI	RightCI	Reference
qFSPL-2-1	2	36c02006	36c02007	1005213	1737807	2020	4.75	0.52	−2.11	25.5	27.5	[13]
qFSPL-2-1	2	36c02006	36c02007	1005213	1737807	2022	5.03	0.52	−2.68	26.5	27.5	
qFSPL-2-2	2	36c02028	36c02029	5593598	5803681	2019	8.19	2.32	−2.79	68.5	71.5	[21]
qFSPL-9-1	9	36c09057	36c09058	9947171	10828881	2022	4.31	0.61	−2.51	135.5	137.5	[12]
qFSPL-13-1	13	36c13063	36c13064	24629868	25640572	2019	3.09	0.60	−0.48	153.5	157.5	
qFSPL-15-1	15	36c15006	36c15007	2413555	2739374	2019 *	10.02	2.94	−1.70	373.5	374.5	
qFSPL-15-1	15	36c15006	36c15007	2413555	2739374	2020 *	3.38	0.60	−2.08	373.5	375.5	
qFSPL-15-1	15	36c15006	36c15007	2413555	2739374	2021 *	3.55	0.40	−2.35	373.5	374.5	
qFSPL-15-1	15	36c15006	36c15007	2413555	2739374	2022 *	8.03	0.59	−2.63	373.5	375.5	
qFSPL-16-1	16	36c16003	36c16004	972045	1294931	2022	3.64	0.50	−2.73	7.5	10.5	
qFSPL-16-2	16	36c16036	36c16027	14760699	15097660	2020	2.62	1.26	−2.02	14.5	15.5	[12]
qFSPL-16-2	16	36c16036	36c16027	14760699	15097660	2021	3.03	0.43	−2.54	14.5	17.5	
qFSPL-17-1	17	36c17019	36c17020	9876026	11105395	2019	3.65	2.27	−1.71	97.5	99.5	[12]
qFSPL-17-2	17	36c17083	36c17082	38236561	39149223	2019	5.41	2.79	−1.74	88.5	89.5	
qFSPL-19-1	19	36c19028	36c19027	8793760	9010938	2022	2.52	0.10	−2.44	151.5	153.5	

* represent this QTL is detected in all four environments.

**Table 4:** Physicochemical property analysis of *Glyma.15G034100* and *Glyma.15G034200.*

Gene	Haplotype	Formula	Estimated Half-Life	Theoretical pI	Extinction Coefficients	Instability Index	Grand Average of Hydropathicity
*Glyma.15G034100*	CDS1	C2358H3660N588O649S18	30 h	5.23	1.746	47.39	0.171
	CDS2	C2357H3660N586O649S18	30 h	5.17	1.748	46.87	0.174
*Glyma.15G034200*	CDS1	C1436H2187N389O436S13	30 h	6.33	1.608	42.68	−0.459
	CDS2	C1437H2187N389O435S13	30 h	6.33	1.608	44.83	−0.462
	CDS3	C1438H2191N389O435S13	30 h	6.33	1.607	44.87	−0.444

## Data Availability

The original contributions presented in the study are included in the article/Supplementary Materials, further inquiries can be directed to the corresponding authors.

## Supplementary Materials

The following supporting information can be downloaded at: https://www.mdpi.com/article/10.3390/plants15060966/s1, Table S1. Summary of detected epistatic QTLs controlling four-seed pod number in the lower part of RIL3613 mapping population of soybean. Table S2. Genes in the interval of qFSPL-15-1 from Phytozome website. Table S3. Amino acid variation of candidate genes.

## Abbreviations

The following abbreviations are used in this manuscript:

FSPN	four-seed pod number
FSPL	four-seed pod lower part
